# Multilevel Associations Between Outlet Characteristics, Contextual Factors and Firearm Violence at On‐Premise Alcohol Outlets in the United States

**DOI:** 10.1111/dar.70111

**Published:** 2026-02-11

**Authors:** Brady Bushover, Leah E. Roberts, Christina A. Mehranbod, Christopher N. Morrison

**Affiliations:** ^1^ Department of Epidemiology, Mailman School of Public Health Columbia University New York New York USA; ^2^ Department of Epidemiology and Preventive Medicine, School of Public Health and Preventive Medicine Monash University Melbourne Australia

**Keywords:** alcohol outlets, firearm policy, firearm violence

## Abstract

**Introduction:**

Firearm violence is a major public health concern in the United States (US). Alcohol use and features of the alcohol environment, including outlet type and alcohol control policies, are factors known to increase the risk of firearm violence. On‐premise alcohol outlets, where alcohol is sold and consumed on site, may influence firearm violence risk through modifications in the physical environment, social processes and policy contexts.

**Methods:**

We conducted a cross‐sectional study examining the association between multilevel characteristics of on‐premise alcohol outlets and other social ecological conditions and shooting incidents across 69 US cities from 2022–2023. Shooting incidents were spatially linked to outlets within a 10‐m buffer. A multivariable generalised linear mixed model with a logit link assessed associations between outlet, neighbourhood, and state factors and the odds of a shooting incident.

**Results:**

The 19,472 on‐premise outlets experienced 290 shooting incidents during the study period. Compared with bars, restaurants (OR = 0.58; 95% CI 0.42, 0.80) and other outlets (OR = 0.58; 95% CI 0.34, 1.00) had lower odds of experiencing a shooting. Greater neighbourhood socioeconomic advantage (per 1 SD increase: OR = 0.64; 95% CI 0.56, 0.74) and stronger state‐level firearm laws (per 1 SD increase: 0.72; 95% CI 0.53, 0.97) were also found to be negatively associated with shooting incidents.

**Discussion and Conclusions:**

Findings align with prior research linking stronger firearm laws, neighbourhood advantage and outlet type to reduced firearm violence risk. Multilevel prevention strategies addressing both structural and outlet‐specific factors may help reduce firearm violence in on‐premise alcohol outlets.

## Key Point Summary

- Non‐bar on‐premise alcohol outlets (restaurants and other types of on‐premise alcohol outlets), greater neighbourhood socioeconomic advantage and stronger firearm laws were associated with decreased odds of a shooting incident at on‐premise alcohol outlets.
- Findings align with prior evidence linking firearm policy strength and neighbourhood conditions to firearm violence risk.
- Extends prior work by showing how firearm policies and neighbourhood disadvantage intersect with local alcohol outlet environments.

## 1 Introduction

Firearm violence is a major public health issue in the United States (US) that contributes substantially to health‐, social‐ and economic‐related burdens [1]. Alcohol use and features of the alcohol environment are established risk factors for firearm violence [2]. On‐premise alcohol outlets, licensed establishments where alcoholic beverages are sold for on‐site consumption, represent an important setting for understanding and preventing firearm violence.

Routine Activity Theory suggests that violent events occur when motivated offenders, suitable targets and a lack of capable guardianship converge in time and space [3]. Consistent with this framework, firearm violence and other crimes are highly concentrated at microgeographic units such as street segments and specific places [4, 5]. Alcohol outlets can create these conditions by increasing social density, extending hours of activity and reducing inhibitions for individuals who consume alcohol [6]. Prior studies have examined outlet type and broader neighbourhood‐ and state‐level correlates of violence. Research consistently links alcohol outlet density to higher rates of violent crime and some previous work has shown that bars often pose a greater risk than other on‐premise outlet types, such as restaurants [7, 8]. Studies have also shown associations between neighbourhood disadvantage and violence, and between stronger alcohol and firearm policies and lower firearm violence [2, 9]. However, most prior studies rely on aggregate geographic measures and not outlet‐specific characteristics. Outlet‐level analyses at microgeographic scales remain limited, largely due to data constraints.

This study aimed to investigate the associations between on‐premise alcohol outlet characteristics, contextual factors and the odds of a shooting incident occurring at these outlets in large US cities.

## 2 Methods

### 2.1 Setting

This study included US Census‐Designated Places with populations of 300,000 or more and available, geolocated shooting incident data, resulting in 69 study cities.

### 2.2 Measures

We obtained outlet‐level data for on‐premise alcohol outlets from the Data Axle US Businesses database [10]. Businesses containing a North American Industry Classification System code of 7224 are categorised as ‘drinking places’ [11], which includes any establishment that sells alcohol for on‐premise consumption. The 2023 American Community Survey provided demographic data for census tracts [12], which functioned as proxies for outlet neighbourhoods. We acquired state‐level data on alcohol‐related laws for on‐premise outlets from established legal and policy databases [13, 14, 15, 16]. State‐level firearm law data were collected from Everytown for Gun Safety [17].

At the outlet level, size was derived from a principal component analysis of outlet characteristics. Alcohol outlet density was calculated as the median distance to the five nearest on‐premise alcohol outlets. Outlet type was categorised as bar, restaurant or other using North American Industry Classification System codes. Neighbourhood‐level covariates, including population size, median age, percent vacant housing and the Index of Concentration at the Extremes (ICE) for racialised economic segregation [18], were measured at the census tract level. State‐level alcohol policies were represented by a constructed score for strength of laws related to on‐premise alcohol outlets. The strength of firearm laws was measured using the Everytown for Gun Safety Gun Law Rankings.

**FIGURE 1:**
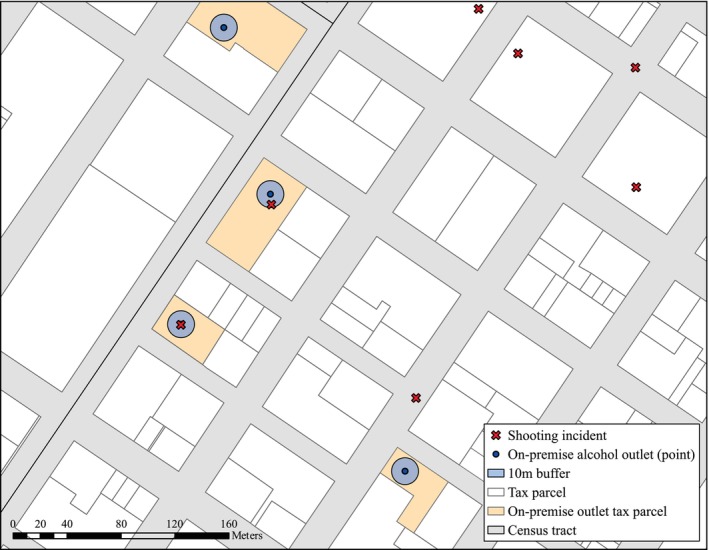
Example of spatial join procedure of shooting incidents to on‐premise alcohol outlets.

We retrieved shooting incidents from the Gun Violence Archive for 2022–2023 [19]. Each incident location was geocoded and then clipped to the administrative boundaries of included cities. We spatially joined shooting incidents to alcohol outlets using 10‐m radial buffers, an approach we have used previously [20] that minimises double counting due to overlapping buffers and accords with the geocoding procedures for both shootings and outlets. Outlets were dichotomised to indicate whether they experienced any shooting incidents during the study period. Figure 1 displays an example of this spatial joining process.

### 2.3 Statistical Analysis

#### 2.3.1 Principal Components Analysis—Outlet Size

The US Businesses database contains numerous variables to describe each outlet. To reduce dimensionality, we performed a principal components analysis (PCA). Categorical variables were encoded prior to PCA. The PCA was performed using the prcomp function in R (version 4.4.1) [21]. Data were centred and scaled before analysis to achieve similar scales across all independent variables. The number of principal components to retain was determined based on the explained variance ratio. The first principal component (PC1) explained 76.7% of the variance, while the first two principal components (PC1 and PC2) explained a cumulative variance of 90.6%. PC1 comprised the number of employees, outlet square footage and sales volume.

#### 2.3.2 Mixed‐Effects Logistic Regression

We used a multivariable generalised linear mixed model (GLMM) with a logit link using the lme4 R package [22] to examine the associations between outlet characteristics, contextual factors, and the odds of a shooting incident, controlling for other outlet‐, neighbourhood‐ and state‐level factors. The model included a random intercept for city to account for clustering of outlets within cities. Clustering within cities was quantified using the intraclass correlation coefficient from an unconditional model. Fixed effects included outlet size, executive gender, outlet type, neighbourhood demographics and state‐level alcohol and firearm policy variables as described above. Because shooting incidents were rare at the outlet level and a small proportion of outlets experienced any shooting incidents during the study period, with very few experiencing more than one event, the outcome was modelled as a binary indicator of whether an outlet experienced at least one shooting. All continuous covariates were centred at the mean and scaled by one standard deviation (SD) prior to analysis. Model fit was summarised using marginal and conditional *R* ^2^ values for generalised linear mixed models [23].

We conducted two sensitivity analyses to address concerns regarding spatial dependencies and the use of a too‐narrow buffer: one utilising a spatial lag term as an additional fixed‐effect covariate and another using a 50‐m radial buffer for evaluating the incidents' proximity to outlets.

## 3 Results

There were 19,472 on‐premise alcohol outlets across the 69 study cities, of which 9684 (49.7%) were bars and 7740 (39.7%) were restaurants. From 2022–2023, 49 (71.0%) cities had an outlet that experienced a shooting incident. Across these outlets, there were a total of 290 shooting incidents at 257 unique outlets. Table 1 provides the study cities with counts of on‐premise outlets and shooting incidents.

**TABLE 1:** Population and counts of on‐premise alcohol outlets and shooting incidents occurring at on‐premise alcohol outlets by study city, 2022–2023.

City	Population	On‐premise outlets	Shooting incidents
New York, NY	8,804,190	2126	22
Los Angeles, CA	3,898,747	867	3
Chicago, IL	2,746,388	929	13
Houston, TX	2,304,580	1056	20
Phoenix, AZ	1,608,139	421	0
Philadelphia, PA	1,603,797	488	21
San Antonio, TX	1,434,625	483	7
San Diego, CA	1,386,932	537	0
Dallas, TX	1,304,379	444	4
San Jose, CA	1,013,240	151	0
Austin, TX	961,855	424	12
Jacksonville, FL	949,611	217	3
Fort Worth, TX	918,915	245	6
Columbus, OH	905,748	354	15
Indianapolis, IN	887,642	289	6
Charlotte, NC	874,579	327	10
San Francisco, CA	873,965	554	3
Seattle, WA	737,015	452	1
Denver, CO	715,522	452	1
Washington, DC	689,545	348	5
Nashville, TN	689,447	293	6
Oklahoma City, OK	681,054	201	6
El Paso, TX	678,815	175	1
Boston, MA	675,647	273	1
Portland, OR	652,503	569	2
Las Vegas, NV	641,903	268	0
Detroit, MI	639,111	218	5
Memphis, TN	633,104	151	13
Baltimore, MD	585,708	296	5
Milwaukee, WI	577,222	312	6
Albuquerque, NM	564,559	127	0
Tucson, AZ	542,629	173	1
Fresno, CA	542,107	94	0
Sacramento, CA	524,943	156	0
Kansas City, MO	508,090	225	4
Mesa, AZ	504,258	89	0
Atlanta, GA	498,715	295	16
Omaha, NE	486,051	299	1
Colorado Springs, CO	478,961	182	0
Raleigh, NC	467,665	165	4
Long Beach, CA	466,742	132	1
Virginia Beach, VA	459,470	36	0
Miami, FL	442,241	223	2
Oakland, CA	440,646	130	1
Minneapolis, MN	429,954	209	15
Tulsa, OK	413,066	107	0
Bakersfield, CA	403,455	73	0
Wichita, KS	397,532	122	2
Arlington, TX	394,266	95	1
Louisville, KY	386,884	78	0
Aurora, CO	386,261	85	0
Tampa, FL	384,959	232	2
New Orleans, LA	383,997	372	14
Cleveland, OH	372,624	201	1
Honolulu, HI	350,964	140	1
Anaheim, CA	346,824	84	0
Lexington, KY	322,570	113	2
Stockton, CA	320,804	32	1
Corpus Christi, TX	317,863	107	1
Henderson, NV	317,610	94	0
Riverside, CA	314,998	47	0
Newark, NJ	311,549	46	0
St. Paul, MN	311,527	92	5
Santa Ana, CA	310,227	30	0
Cincinnati, OH	309,317	184	6
Irvine, CA	307,670	33	0
Orlando, FL	307,573	190	2
Pittsburgh, PA	302,971	229	6
St. Louis, MO	301,578	231	4

Table 2 presents the results of the logistic regression model. The unconditional model intraclass correlation coefficient was 0.19, indicating meaningful city‐level clustering. On‐premise outlet type was associated with shooting incidents. Compared to bars, restaurants had 42% lower odds of experiencing a shooting incident (odds ratio [OR] = 0.58; 95% confidence interval [CI] 0.42, 0.80), and outlets in the ‘other’ category similarly had lower odds (OR = 0.58; 95% CI 0.34, 1.00). At the neighbourhood level, greater socioeconomic advantage, as measured by the ICE score for racialised economic segregation (per 1 SD increase), was associated with substantially lower odds of a shooting incident (OR = 0.64; 95% CI 0.56, 0.74). At the state level, stronger firearm law scores (per 1 SD increase) were associated with reduced odds of a shooting incident (OR = 0.72; 95% CI 0.53, 0.97). Other outlet‐, neighbourhood‐ and state‐level characteristics, including outlet size, alcohol outlet density, executive gender, population size, median age, percent vacancy and on‐premise alcohol outlet laws, showed estimated odds ratios with confidence intervals that included the null value of 1. The mixed‐effects model explained a meaningful proportion of variability in shooting risk, with a marginal *R* ^2^ of 0.18 for fixed effects and a conditional R^2^ of 0.26 when accounting for between‐city variation. Results of the sensitivity analyses indicated that the findings remained robust when incorporating a spatial lag term as an additional fixed‐effect covariate in the model (Table S1) and when using a 50‐m buffer (Table S2).

**TABLE 2:** Results from multivariable generalised linear mixed model with logit link.

Variable	OR	95% CI	*p*
Outlet size^a^	0.98	(0.85, 1.15)	0.793
Alcohol outlet density^a^	1.01	(0.87, 1.17)	0.895
Executive gender			
Female	Ref.	Ref.	Ref.
Male	0.90	(0.62, 1.29)	0.573
Unknown	0.97	(0.66, 1.42)	0.876
On‐premise outlet type			
Bar	Ref.	Ref.	Ref.
Restaurant	**0.58**	**(0.42, 0.80)**	**< 0.001**
Other	0.58	(0.34, 1.00)	0.056
Population^a^	0.95	(0.81, 1.12)	0.535
ICE^a^	**0.64**	**(0.56, 0.74)**	**< 0.001**
Age^a^	0.88	(0.76, 1.03)	0.100
Percent vacant	1.84	(0.34, 9.90)	0.478
Alcohol law score^a^	1.24	(0.97, 1.59)	0.088
Firearm law score^a^	**0.72**	**(0.53, 0.97)**	**0.033**

Abbreviations: CI, confidence interval; ICE, Index of Concentration at the Extremes; OR, odds ratio.

^a^ Standardised (mean = 0, SD = 1) prior to analysis.

## 4 Discussion

In this multilevel analysis of on‐premise alcohol outlets across large US cities, we identified outlet‐, neighbourhood‐, and state‐level factors associated with reduced shooting risk. These findings are consistent with previous research showing that stronger firearm policies and reduced neighbourhood disadvantage are protective against violence [2, 9], and that bars are associated with higher risks for violence than other types of on‐premise alcohol outlets [8].

While firearm policies and neighbourhood disadvantage are well‐documented determinants of firearm violence, our study examines how they intersect with local alcohol environments. Stronger firearm policies and reduced neighbourhood disadvantage may create protective conditions against firearm violence risk, along with certain outlet‐level features. These findings highlight the value of multilevel prevention approaches combining structural factors with targeted interventions at high‐risk commercial locations. Although outlet size was found not to be statistically significantly associated with shooting incidents in our analysis, prior research suggests that larger venues may facilitate higher patron volumes and more complex management challenges, potentially influencing risk [24]. The lack of association in this study may reflect limitations inherent to the design, measurement constraints or insufficient variation in outlet size across settings.

Some limitations should be noted. First, the cross‐sectional nature of this analysis precludes causal inference. The observed associations could reflect unmeasured confounding or reverse causation. Also, the data from which outlet characteristics were derived may contain reporting errors. While the Gun Violence Archive has been previously validated for large US cities [25], shooting incidents may have been undercounted or misclassified. Finally, since this analysis was limited to large cities, the findings may not generalise to smaller areas or rural communities.

## 5 Conclusions

This study adds evidence that stronger firearm legislation, greater neighbourhood socioeconomic advantage, and non‐bar outlet types are associated with reduced firearm violence risk in alcohol‐serving environments. By focusing on individual outlets rather than only aggregate geographic measures, this study offers more actionable insights for local policymakers, enforcement agencies and licensing boards.

These findings emphasise the importance of multilevel violence prevention approaches that address both place‐specific and structural risk factors. Policies strengthening firearm regulations, reducing concentrated disadvantage and tailoring licensing or operational requirements for high‐risk outlet types may reduce firearm violence at on‐premise alcohol outlets. Future research that combines detailed outlet‐level data with longitudinal designs will be critical for identifying modifiable characteristics and informing targeted, evidence‐based interventions.

## Author Contributions

All listed authors contributed to the conceptualisation and objectives of the study. B.B. and C.N.M. developed the study design and procedures. B.B. conducted the analysis and drafted the manuscript. C.N.M. provided feedback and ongoing oversight throughout study conceptualisation, data analysis and interpretation. All authors contributed to manuscript editing and critically revised the manuscript for important intellectual content, approved the final version for publication, and agree to be accountable for all aspects of the work. Each author certifies that their contribution to this work meets the standards of the International Committee of Medical Journal Editors.

## Funding

This work was supported by grant K18HD117390 from the Eunice Kennedy Shriver National Institute of Child Health and Human Development of the National Institutes of Health. The findings and conclusions in this paper are those of the authors and do not necessarily represent the views of the funders.

## Conflicts of Interest

The authors declare no conflicts of interest.

## Supporting information


**Table S1:** Results from sensitivity analysis with included spatial lag term.
**Table S2:** Results from sensitivity analysis utilising 50 m radial buffers.

## Data Availability

The data that support the findings of this study are available from Data Axle Reference Solutions and the Gun Violence Archive. Restrictions apply to the availability of these data, which were used under licence for this study. Data are available from https://www.data‐axle.com/platforms‐products/reference‐solutions/ with the permission of Data Axle Reference Solutions, and from https://www.gunviolencearchive.org/ with the permission of the Gun Violence Archive. The data that support the findings of this study are also available from the US Census Bureau at https://data.census.gov/.
