# Supplementary material for: Multilevel Associations Between Outlet Characteristics, Contextual Factors and Firearm Violence at On‐Premise Alcohol Outlets in the United States
Source: Drug Alcohol Rev. 2026 Feb 11;45(2):e70111. doi: 10.1111/dar.70111 (PMC12892834; doi:10.1111/dar.70111)
Supplement: Supplementary file 1 — Table S1: Results from sensitivity analysis with included spatial lag term. Table S2: Results from sensitivity analysis utilising 50 m radial buffers. [file DAR-45-0-s001.docx]

**Table S1. Results from sensitivity analysis with included spatial lag term.**

| **Variable** | **OR** | **95% CI** | **p-value** |
| --- | --- | --- | --- |
| Outlet size^†^ | 1.00 | (0.86, 1.16) | 0.969 |
| Alcohol outlet density^†^ | 0.94 | (0.80, 1.10) | 0.478 |
| **Spatial lag term** | **1.02** | **(1.01, 1.03)** | **<0.001** |
| Executive gender |  |  |  |
| Female | Ref. | Ref. | Ref. |
| Male | 0.90 | (0.63, 1.29) | 0.570 |
| Unknown | 0.98 | (0.67, 1.43) | 0.925 |
| On-premise outlet type |  |  |  |
| Bar | Ref. | Ref. | Ref. |
| **Restaurant** | **0.58** | **(0.42, 0.80)** | **<0.001** |
| **Other** | **0.59** | **(0.34, 0.98)** | **0.043** |
| Population^†^ | 0.96 | (0.82, 1.12) | 0.611 |
| **ICE^†^** | **0.70** | **(0.61, 0.81)** | **<0.001** |
| Age^†^ | 0.88 | (0.76, 1.02) | 0.082 |
| Percent vacant | 1.73 | (0.33, 9.02) | 0.515 |
| Alcohol law score^†^ | 1.16 | (0.92, 1.46) | 0.206 |
| **Firearm law score^†^** | **0.72** | **(0.55, 0.95)** | **0.020** |

^†^standardised (mean = 0, SD = 1) prior to analysis

CI, confidence interval; ICE, Index of Concentration at the Extremes; OR, odds ratio.

**Table S2. Results from sensitivity analysis utilizing 50 m radial buffers.**

| **Variable** | **OR** | **95% CI** | **p-value** |
| --- | --- | --- | --- |
| Outlet size^†^ | 1.00 | (0.94, 1.07) | 0.937 |
| **Alcohol outlet density^†^** | **0.73** | **(0.66, 0.80)** | **<0.001** |
| Executive gender |  |  |  |
| Female | Ref. | Ref. | Ref. |
| Male | 0.98 | (0.83, 1.15) | 0.784 |
| Unknown | 1.14 | (0.96, 1.35) | 0.147 |
| On-premise outlet type |  |  |  |
| Bar | Ref. | Ref. | Ref. |
| **Restaurant** | **0.69** | **(0.60, 0.79)** | **<0.001** |
| **Other** | **0.60** | **(0.47, 0.75)** | **<0.001** |
| Population^†^ | 0.93 | (0.87, 1.01) | 0.071 |
| **ICE^†^** | **0.65** | **(0.61, 0.69)** | **<0.001** |
| Age^†^ | 0.95 | (0.89, 1.02) | 0.149 |
| **Percent vacant** | **7.09** | **(3.49, 14.4)** | **<0.001** |
| Alcohol law score^†^ | 1.08 | (0.91, 1.27) | 0.391 |
| Firearm law score^†^ | 1.00 | (0.83, 1.21) | 0.991 |

^†^standardised (mean = 0, SD = 1) prior to analysis.

CI, confidence interval; ICE, Index of Concentration at the Extremes; OR, odds ratio.
